# Multifunctional Device based on phosphor-piezoelectric PZT: lighting, speaking, and mechanical energy harvesting

**DOI:** 10.1038/s41598-017-18571-9

**Published:** 2018-01-10

**Authors:** Sunghoon Lee, Taewook Kang, Wunho Lee, Mohammad M. Afandi, Jongho Ryu, Jongsu Kim

**Affiliations:** 1 R&D Business Lab, Hyosung Corporation, Anyang, 431-080 Republic of Korea; 2 Interdisciplinary Program of LED Convergence, Pukyong National University, Busan, 608-737 Republic of Korea; 3 Department of Display and Science Engineering, Pukyong National University, Busan, 608-737 Republic of Korea

## Abstract

We demonstrated the tri-functional device based on all powder-processing methods by using ZnS powder as phosphor layer and piezoelectric material as dielectric layer. The fabricated device generated the electroluminescent (EL) light from phosphor and the sound from piezoelectric sheet under a supply of external electric power, and additionally harvested the reverse-piezoelectric energy to be converted into EL light. Under sinusoidal applied voltage, EL luminances were exponentially increased with a maximum luminous efficiency of 1.3 lm/W at 40 V and 1,000 Hz, and sound pressure levels (SPLs) were linearly increased. The EL luminances were linearly dependent on applied frequency while the SPLs showed the parabolic increase behavior below 1,000 Hz and then the flat response. The temperature dependence on EL luminances and SPLs was demonstrated; the former was drastically increased and the latter was slightly decreased with the increase of temperature. Finally, as an energy harvesting application, the piezoelectric-induced electroluminescence effect was demonstrated by applying only mechanical pressure to the device without any external electric power.

## Introduction

The powder electroluminescence (EL) device consists of a transparent electrode on substrate, a phosphor layer and a dielectric layer dispersed in an organic binder and a rear electrode^1^. The EL phenomenon is attributed to phosphor excitation by hot electrons injected from dielectric layer to phosphor layer when a high electric field in order of 10^6^–10^7^ V/m is applied to phosphor layer^2^. The most of ZnS-based phosphors are commercially available for powder EL: blue ZnS:Cu, Cl, green ZnS:Cu, Al and orange ZnS:Cu, Mn. The powder EL devices have non-glare uniform light emission, thin profile of about 200 µm and low power consumption of about 30 W/m^2^ for a luminance of about 100 cd/m^2^ ^3^. They are used for liquid crystal display (LCD) backlight, and architectural and decorative lighting. Special EL application is the display panel for very harsh environments such as space modules and fighting vehicles due to high thermal stability over the temperature range of −50/ + 100 °C.

The recent need for EL device is the multi-functionality. There are some researches on bi-functional devices on the base with phosphor and piezoelectric material: dual emission of sound and light in sound-generating (acoustic) EL device based on piezoelectric layer, i.e., EL speaker, Y. Zhang *et al*. reported the light-ultrasound dual emission in ZnS:Mn/PMN-PT [Pb(Mg_1/3_Nb_2/3_)O_3__−x_PbTiO_3_] thin film structure^4^, and T. Minami *et al*. announced the sound-emitting EL emission in ZnS:Mn/BaTiO_3_ thin film structure^5^. In addition, piezoelectric-driven electroluminescence without electric power was demonstrated through a coupling of phosphor and piezoelectric material^6,7^. However, to our knowledge, there is no report on tri-functional and powder-based processed devices: the light emission from the phosphor and simultaneously the sound emission from the piezoelectric as well as the piezo-electric-luminescence (PEL) by energy harvesting of external mechanical strain or vibration to be converted into light energy.

In this study, we demonstrated the tri-functional device based on all powder-processing methods by using ZnS powder as phosphor layer and piezoelectric material as dielectric layer. The device consists of silver nanowires as top electrode, and ZnS:Cu, Al phosphor screen-printed on high piezoelectric material, i.e., lead zirconate titanate (PZT-4) ceramic sheet, which is chosen due to its high dielectric and piezoelectric properties as well as its most cost-effectiveness of other higher dielectric-piezoelectric PZT families such as PZT-5A and PZT-5H^8–10^. The fabricated device generated the light from the phosphor and the sound from PZT sheet under an external electric power, and additionally harvested the mechanical energy such as external strain or vibration to be converted into electric energy or light energy. For the feasibility test of the device, the temperature dependences on the sound pressure level (SPL) and the light luminance were measured; as a result, the former was slightly decreased and the latter was drastically increased with the increase of temperature. Finally the piezoelectric-induced electroluminescence effect was demonstrated by applying the external pressure to the device without any electric power.

## Results

The tri-functional device based on ZnS-phosphor and PZT layers was demonstrated. The fabricated device generated the EL light from the phosphor and the sound from the piezoelectric sheet under a supply of external electric power, and additionally harvested the reverse-piezoelectric energy harvesting mechanical to be converted into EL light. The high *k* value (~1650) of piezoelectric layer is attributed to high EL luminance up to 127 cd/m^2^ at 250 V and 1,000 Hz. The maximum luminous efficiency of 1.3 lm/W was achieved at 40 V and 1,000 Hz. The high *d*_33_ value (~400 pC/N) of piezoelectric layer resulted in high SPL up to 88 dB at 100 V and 3,600 Hz. The EL luminance was exponentially dependent on the applied voltage while the SPL was linearly dependent on it, with the same threshold voltage of 10 V. The EL luminance was linearly increased with increasing the applied frequency while the SPL showed the parabolic increase phenomenon below 1,000 Hz and then the flat response. The temperature-dependent EL luminance reached the maximum point at the Curie temperature of PZT sheet of 230 °C, while the SPL was slightly decrease from 73 dB (25 °C) to 65 dB (250 °C). In addition, our device produced power-free EL light by converting the mechanical pressure into the optical energy.

## Discussion

**Figure 1:**
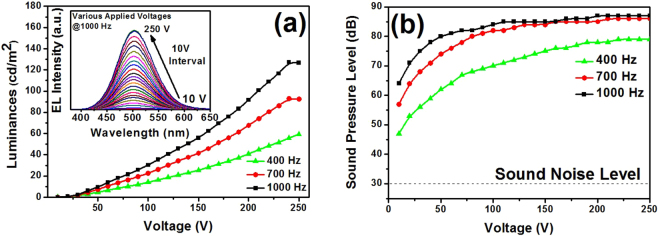
Luminance-voltage (L-*V*) characteristics (**a**) with the inset on their EL spectra, and sound pressure level-voltage (SPL-*V*) characteristics (**b**).

Figure 1(a) shows EL spectra of EL device driven by a sinusoidal power at various applied voltages for the fixed frequency of 1,000 Hz. The peak wavelength is 503 nm and the half width is 80 nm. The CIE color coordinate are x = 0.2477 and y = 0.4411. As the voltage increases, the EL intensities are exponentially increased without spectral variation. The EL emission peak is assigned to the transition from the donor level of Al^3+^ ions on Zn^2+^ sites to the acceptor level of Cu^+^ ions on Zn^2+^ sites, i.e., donor-acceptor recombination^11–13^. The threshold voltage (*V*_*th*_) was determined about 10 V according to the interpolation of x-intercept in voltage-luminance curve. The electric field inside the phosphor layer at V_th_ is calculated as 2.5 × 10^5^ V/m (~10 V/40 μm), which is comparable to the previously reported value. The maximum luminance of 127 cd/m^2^ was obtained at 250 V and 1,000 Hz. In conventional powder EL devices, the luminance is given by the equation (1)^14^:

1 $$L={L}_{0}{e}^{(-b{V}^{-1/2})}$$

where *L*_0_ and *b* are constants, which depend on the particle size of phosphor, the concentration of activator, the thickness of light emitting layer, and the dielectric constant of organic binder. The equation reveals that the logarithm of *L* is proportional to *V* ^*−1/*2^; luminance (*L*) versus applied voltage (*V* ^*−1/*2^) is well fitted with the above equation, as plotted in Fig. 1(a). It suggests that the EL mechanism follows that of well-known ZnS-based powder EL device; the ballistic injection of electrons accumulated at dielectric surface caused by electric breakdown of phosphor layer^2,3^.

Figure 1(b) shows SPL versus input voltage of the fabricated EL device. It shows the good linearity at lower voltages, and then the saturation behavior at higher voltages than about 100 V. The SPL is a function of input voltage *V* as the equation (2)^15^:

2 $$SPL\propto 20\,\mathrm{log}\,(V)$$

First, the lower-voltage linearity is attributed to the linear dependence of voltage on piezoelectric displacement, which is directly related to the amplitude of sound wave which is generated by the piezoelectric ceramics^16^. Second, the SPL saturation behavior at higher voltages (the maximum SPL of about 86 dB at 1,000 Hz) is attributed to the physical limitation of piezoelectric displacement^17^.

**Figure 2:**
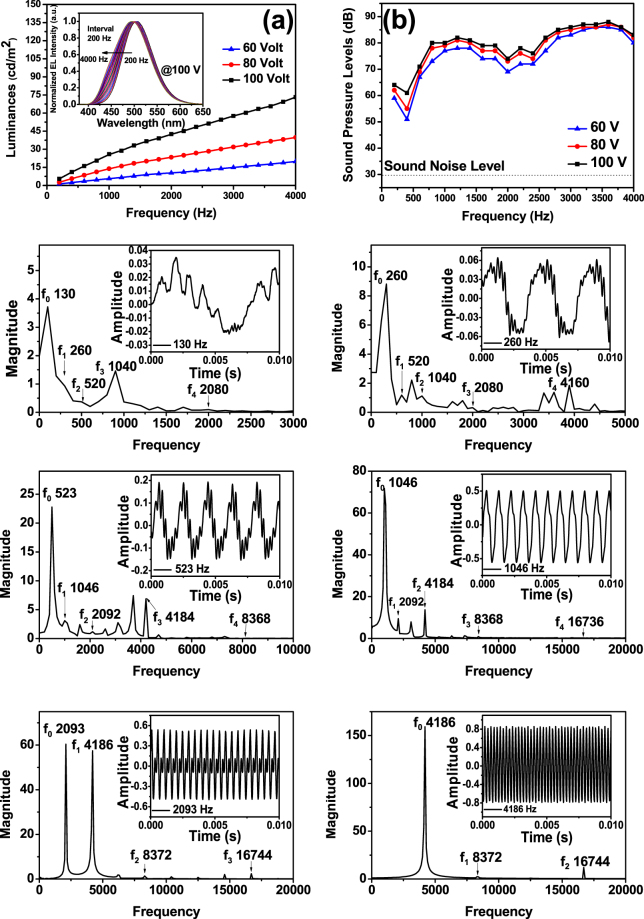
Luminance-frequency (L-*f*) characteristics (**a**) with the inset on their EL spectra, sound pressure level-frequency (SPL-*f*) characteristics (**b**), and sound signal outputs in frequency domain with the inset on their time-domain: 130 Hz, 260 Hz, 523 Hz, 1,046 Hz, 2,093 Hz and 4,186 Hz (**c**).

Figure 2(a) displays EL spectra at various frequencies up to 4,000 Hz at the fixed voltage of 100 V. The EL spectra are strongly dependent on applied frequencies (508 nm at 200 Hz, 503 nm at 1,000 Hz, and 494 nm at 4,000 Hz), which is consistent with the blueshift behavior of the standard EL device using ZnS phosphor^18,19^. There are two dominated transition channels inside ZnS:Cu, Al phosphor, ignoring both the infrared-energy transitions from conduction band to donor level or acceptor level to valance band; (1) the green-energy transition (503 nm peak) from donor level to acceptor level, and (2) the blue-energy transitions (460 nm peaks) from conduction band to acceptor level and donor level to valance band. At lower frequencies, the donor-acceptor recombination (1) dominates between the donor level of Al^3+^ ions on Zn^2+^ sites and the acceptor level of Cu^+^ ions on Zn^2+^ sites. At higher frequencies, the high-speed excitation provides too short time for electrons on the conduction band to be decayed to the donor level, or for holes on the valance band to fall down to the acceptor level, so the electron-hole population in the donor-acceptor levels decreases so as to quench the donor-acceptor recombination, while the electron-hole population in the conduction-valance bands increases and thus the transition from the conduction band to the acceptor level and the donor level to the valance band dominates (2), as shown in the inset of Fig. 2(a). Also, the EL luminances are linearly increased with increasing frequencies. This trend can be explained in terms of the increasing numbers of excitation of host lattice or Cu-Al recombination with increasing frequency.

Figure 2(b) shows the frequency response of our EL speaker at 100 V. Frequency response of an ideal piezoelectric speaker can be modeled as a single-resonance system^20^. Below the resonance frequency (*f*_*r*_), SPL is proportional to the square of the frequency (~*f* ^2^), Above *f*_*r*_, SPL is approximately independent of the frequency, i.e., the flat response. According to the frequency response, *f*_*r*_ value of our EL speaker is estimated as 1,000 Hz. Below f_r_, the frequency response follows the as-mentioned single-resonance mode: *SPL* ~*f* ^*2*^. Above *f*_*r*_, our EL speaker shows the flat response with the constant SPL of about 85 dB (100 V).

Figure 2(c) shows the sound signal outputs of EL speaker in frequency domain. Each inserted figures show the sound signal outputs of EL speaker in time domain. The sound signal outputs in frequency domain were obtained by Fourier transformation of time-domain spectra. Various applied frequencies with a sinusoidal signal were applied to EL speaker: *C*_3_ = 130 Hz (“Do” of third octave), *C*_4_ = 260 Hz (“Do” of fourth octave), *C*_5_ = 523 Hz (“Do” of fifth octave), *C*_6_ = 1,046 Hz (“Do” of sixth octave), *C*_7_ = 2,193 Hz (“Do” of seventh octave) and *C*_8_ = 4,186 Hz (“Do” of eighth octave) in scientific notation. All sound spectra shows the dominated magnitude at the same sound frequencies with applied frequencies. The second strongest magnitude is observed at the double or the triple of applied frequencies. The signals at the quadruple frequencies are comparatively weak. These signals are attributed to second, third and fourth harmonic oscillations of the vibrational piezoelectric plate.

**Figure 3:**
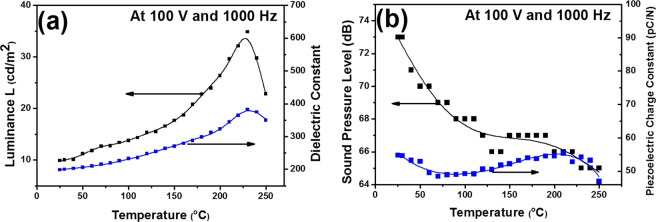
Temperature dependence of luminance (L) and dielectric constant (*k*) (**a**) and sound pressure level (SPL) and piezoelectric charge constants (*d*_33_) (**b**).

Figure 3(a) shows the temperature dependent EL spectra of EL device at sinusoidal voltage of 100 V with frequency of 1,000 Hz. It is well known that photoluminescent intensities are significantly decreased along with temperatures due to thermal quenching effect^21^. On the contrary, EL intensities are drastically increased with temperatures. It is attributed not to the nature of the phosphor (thermal quenching) but the other effect which is the temperature dependence of dielectric constant of PZT layer. In a conventional dielectric material such as PZT, its dielectric constant is reported to be maximized at Curie temperature (230 °C) and then decreased^22^. Ignoring thermal quenching effect of phosphor, the temperature dependence of our EL device using PZT dielectric layer follows that of its dielectric constant (*k*). Finally we can conclude that ZnS/PZT-based EL device is not degraded in the EL intensity even under hot ambient temperature, and it shows higher stability in an extreme operation condition.

Figure 3(b) shows the temperature dependent SPLs of EL speaker at sinusoidal voltage of 100 V and frequency of 1,000 Hz. It is well established that with the increase of temperature the piezoelectric charge constants (*d*_33_) of PZT material are slightly increased and then reached the maximum around its Curie temperature, and then significantly quenched^23^. Therefore the SPL is expected to be increased with increasing temperature. However, our temperature-dependent SPLs of our EL speaker show a slight decreasing trend. This behavior results from the stronger temperature dependence of both air density and sound velocity than that of d_33_ value but also decreased by the increasing of temperature. The SPL of a standard speaker is expressed by the equation (3)^24^:

3 $$\begin{array}{lll}SPL & \propto  & 10\,{log}\,\frac{I}{{I}_{ref}}\propto 20\,log{P}_{m}-\,\mathrm{log}\,\rho \cdot c\\  & \propto  & 20\,{log}\,{d}_{33}-(20 \sim 30)\,{log}\,T\end{array}$$

where *I* is the intensity of sound and *I*_*ref*_ is the intensity of the reference at 101.3 kPa. Moreover, *I = P*_*m*_^2^ */ρ·c*, where *P*_*m*_ is the maximum amplitude of the sound wave which is determined by a piezoelectric displacement as a function of the piezoelectric charge constant (*d*_33_). The *d*_33_ is linearly generated by the applied voltage (*V*), while *ρ* is the air density with the linear dependence on the barometric pressure (*B*) and inversely linear dependence on the air temperature (*T*), and *c* is the sound velocity affected by the ambient temperature (*T*) via the air density (*ρ)*. Therefore, the SPL is more sensitive to the ambient temperature than the piezoelectric *d*_33_ value, so the SPL is slightly decreased with the ambient temperature from 73 dB (25 °C) to 65 dB (250 °C) even though the piezoelectric d_33_ value is increased with increasing the ambient temperature^25^.

**Figure 4:**
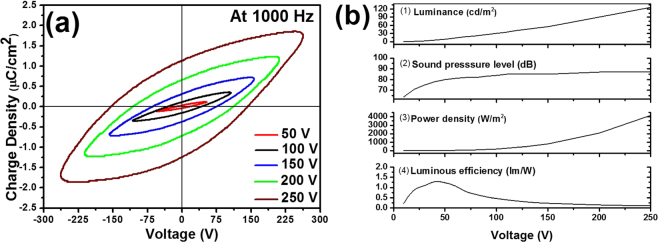
Charge density-voltage (*Q-V*) diagram (**a**) and the luminance-SPL-power density-luminous efficiency-voltage (L-SPL-*P*-*ƞ-V*) characteristics (**b**).

Figure 4 depicts charge density (*Q*) versus voltage (*V*) at the frequency of 1,000 Hz. The *Q–V* diagram is based on a conventional Sawyer-Tower circuit using the sense capacitor with a capacitance of 1 μF at 1,000 Hz. As the voltage increases, the remaining charge densities at the transient voltage of zero are increased from 0.03 µC/cm^2^ at 50 V to 1.13 µC/cm^2^ at 250 V. The charge density reaches the maximum of 1.76 µC/cm^2^ at 250 V in the falling phase of applied voltage. The integrated area (*A*) of the *Q–V* diagram means the power consumption to EL device per cycle. Assuming a diffusive EL emission surface of EL device, the luminous efficiency (*η*) is given by the equation (4)^26^;

4 $$\eta =\frac{\pi \cdot L}{f\cdot A}$$

where *L* is the luminance in the unit of cd/m^2^, and *f* is the frequency in the unit of Hz, and *A* is the encompassed area of *Q-V* diagram in the unit of W/m^2^. At higher voltages in which the higher luminances and SPLs were emitted, the power consumption was extremely increased up to two orders of magnitude (4,100 W/m^2^ at 250 V), and simultaneously the luminous efficiency was significantly decreased (0.10 lm/W at 250 V). The enormous power consumption and the diminutive efficiency are attributed to the worst efficient piezoelectric speaker (about 1,100 W/m^2^ at 30 V)^27^. Nevertheless, at lower voltages below 100 V, the power consumption and efficiency of our EL device was still better and applicable. The maximum luminous efficiency (*η*) is calculated 1.3 lm/W based on the luminance of 6 cd/m^2^ and the power consumption of 14 W/m^2^ at 40 V, which is comparable to the previous result of ZnS-BaTiO_3_ powder EL device^28^.

**Figure 5:**
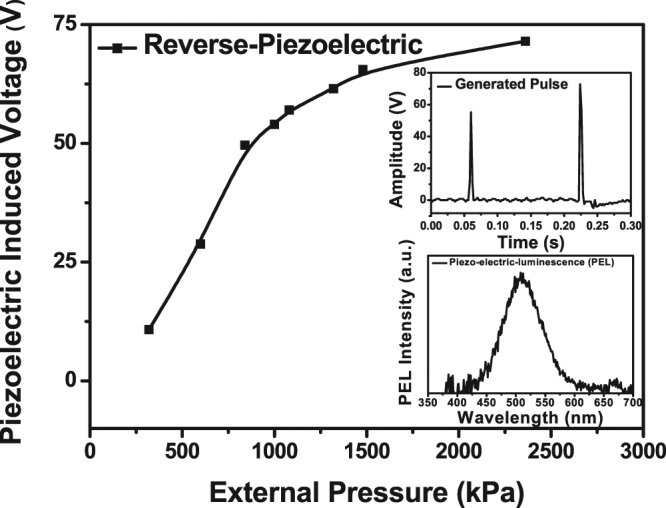
Piezoelectric voltage versus the mechanical pressure (*V*_*g*_ *−p*); the top inset is the voltage pulse waveform, and the bottom is the pressure-driven EL spectrum.

Figure 5 depicts the mechanical pressure (*p*)-induced voltage (*V*_*g*_) at room temperature. The top inset shows the generated pulse voltage waveform, and the bottom inset is the pressure-induced EL spectrum. The mechanical pressure was exerted onto the surface of EL device by dropping various weights. The mechanical pressure generated the pulse voltage with a rise time of 5 millisecond, as seen in the inset of Fig. 5, which causes the EL for the short period. It is the green light with 510 nm peak, as shown in the inset of Fig. 5. As the pressure increases, the generated piezoelectric voltage was linearly increased until it reached 1,000 kPa, and then it was saturated due to the maximum displacement of piezoelectric at that pressure^29^. It indicates that this pressure-induced EL light can be applicable to some applications in energy harvesting device as well as in remote stress sensor under a power-off situation^30^.

## Methods

**Figure 6:**
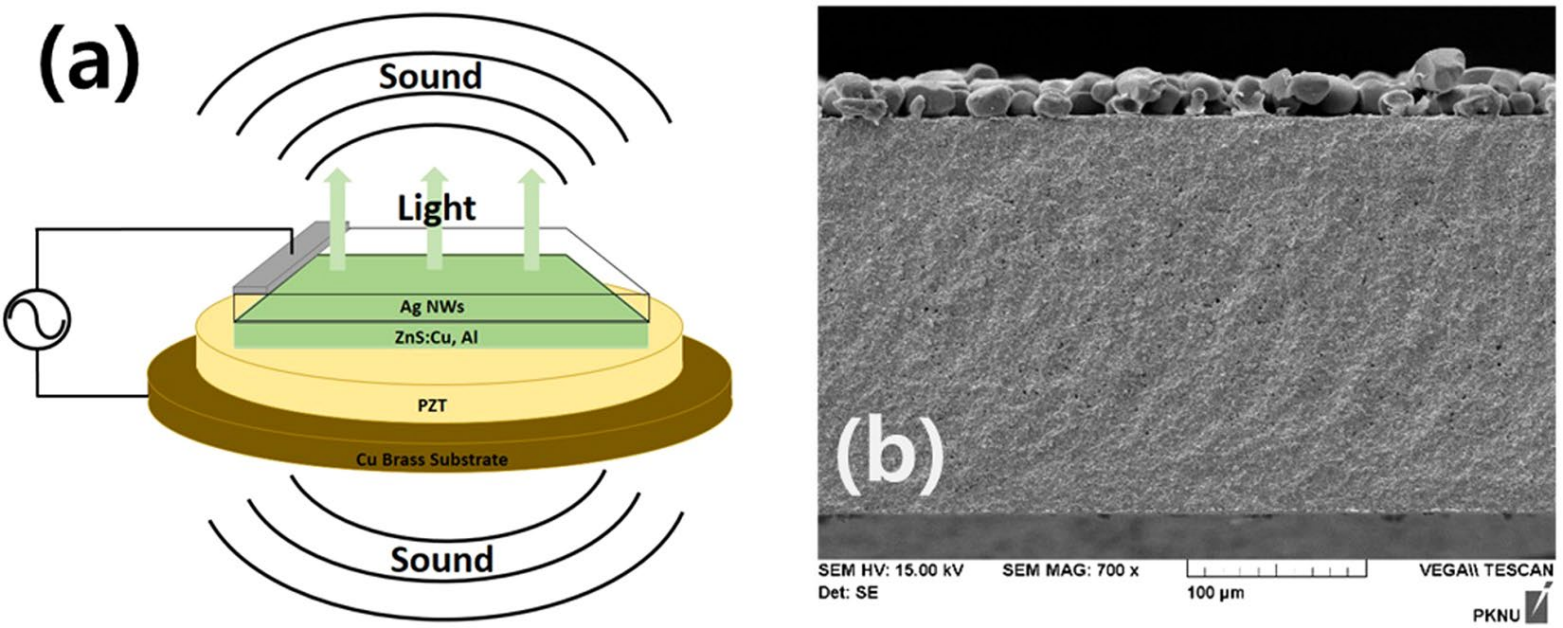
Structure of EL device (**a**) and its SEM cross-section image (**b**).

The commercial ZnS:Cu, Al phosphor powder with a mean particle of 30 µm was used and dispersed in cyan ethyl cellulose as organic binder which had the highest dielectric property of various resin-type binders. The mixture was two times printed on the dielectric PZT sheet by a screen printing method, which was 10 mm by 10 mm in size. In this study, the optimized thickness of the phosphor layer (emissive layer) was 40 μm for lower threshold voltage and high luminous efficiency. In this experiment, the commercial 200 μm-thick PZT circular sheet (Murata) with a diameter of 19 mm was combined with the hard metal substrate: its piezoelectric charge constant (*d*_33_) = 400 pC/N, and its relative dielectric constant (*k*) = 1,650^20^. The commercial silver nanowires were employed as top electrode on the phosphor layer. The silver nanowires solution resolved in deionized water was spin-coated on the ZnS phosphor layer. The coated silver nanowires were dried at 120 °C for 10 minutes. The transparency of coated silver nanowires transparent electrode was measured to be 70% at 450 nm and 80% at 510 nm by using a UV-Vis spectrophotometer (Lambda 40, Perkin Elmer). The sheet resistivity of silver nanowires electrode was 6 Ω/sq by Van Der Pauw method^31,32^. The structure of the fabricated EL device was shown in Fig. 6(a), and its cross-section SEM image showed Fig. 6(b).

The electroluminescent properties of EL device was measured using spectrometer (Konica Minolta, CS-2000). Charge density versus applied voltage (*Q-V*) characteristic of EL device was measured using Sawyer-Tower circuit. The piezoelectric properties of EL device was measured by (IACAS, piezo meter). SPL in dB unit were measured with a distance of one cm between the EL device and the microphone. Time and frequency-domain acoustic spectra were analyzed by a program of Gold Wave Digital Audio Editor. The PEL effect was tested by dropping weights onto the surface of EL device, and the output voltage and the luminescence was recorded.

## Electronic supplementary material

Various Frequencies

Diatonic Frequencies
